# Nutritional Value of Duckweed as Protein Feed for Broiler Chickens—Digestibility of Crude Protein, Amino Acids and Phosphorus

**DOI:** 10.3390/ani13010130

**Published:** 2022-12-29

**Authors:** Johannes Demann, Finn Petersen, Georg Dusel, Manuela Bog, Reindert Devlamynck, Andreas Ulbrich, Hans-Werner Olfs, Heiner Westendarp

**Affiliations:** 1 Faculty of Agriculture and Environmental Sciences, University of Rostock, 18059 Rostock, Germany; 2 Faculty of Agricultural Sciences and Landscape Architecture, University of Applied Sciences Osnabrück, 49090 Osnabrück, Germany; 3 Life Science and Engineering, University of Applied Sciences Bingen, 55411 Bingen am Rhein, Germany; 4 Institute of Botany and Landscape Ecology, University of Greifswald, 17489 Greifswald, Germany; 5 Inagro vzw, 8800 Rumbeke-Beitem, Belgium

**Keywords:** tannin, phytate, animal performance, feed intake, water binding capacity

## Abstract

**Simple Summary:**

An increasing global population will lead to an increased demand for protein and a protein deficit. The production of soy, the most common protein source in animal nutrition, is often associated with deforestation and long transport distances. In this context, duckweed can be considered an alternative protein source for animal nutrition. The term duckweed describes a group of small plants floating on the water surface with sizes up to 1.5 cm. Three different duckweed batches consisting of different species were tested as feed for broiler chickens. They showed different impacts on feed intake, animal growth, and digestibility. Digestibility describes the share of nutrients resorbed within the digestive system. Possible compounds that inhibited the digestibility were identified. The results suggest that the nutritional value of duckweed and its influence on animal performance are variable. Hence, a stable duckweed biomass quality adapted to the requirements of broilers is needed.

**Abstract:**

Duckweed is gaining attention in animal nutrition and is considered as a potential alternative protein source for broiler chickens. In order to evaluate the nutritional value of duckweed, three individual batches were investigated. They consisted of a mixture of *Lemna minuta* and *Lemna minor* (A, 17.5% crude protein), *Spirodela polyrhiza* (B, 24.6% crude protein) and *Lemna obscura* (C, 37.0% crude protein). Treatment diets contained 50% batch A, 50% batch B, and 25, 50 and 75% of batch C. All diets were fed to broiler chickens (Ross 308) from an age of 21 to 27 days. Diets with a share of 50 and 75% of batch C led to decreased feed intake (109.3 and 74.9 g/day, respectively) compared to the control. Standardized ileal digestibility of crude protein and amino acids differed significantly between duckweed batches, at values for methionine between 49.9 and 90.4%. For all amino acids, batch A consistently had the lowest and batch C the highest digestibility. Batches had different tannin contents of 2943, 2890 and 303 mg/kg for batches A, B and C, respectively. The apparent ileal digestibility of phosphorus differed significantly between all batches (50.8–78.9%). Duckweed can be used as a protein feed for broiler chickens. However, a defined and stable biomass composition optimized for the requirements of broiler chickens is needed.

## 1. Introduction

United Nations [1] projections estimate that the global population will increase to 9.7 billion by 2050, leading to an increasing demand for animal protein and a global protein gap [2]. The European Union is already dependent on imports of protein-rich feedstuffs, mainly from America, in the form of soybean meal due to its high protein content and high levels of limiting amino acids [3]. The decoupling of land farming and livestock production causes several environmental and sustainability issues, such as deforestation in South America or nutrient surpluses in areas with intense livestock production [3,4]. However, the growth potential for common plant proteins is limited in the segment of complete feed [3].

In this context, duckweed can be considered an alternative protein source for broiler nutrition. The term duckweed describes a group of flowering aquatic plants including the five genera of *Lemna* L., *Spirodela* Schleid., *Landoltia* Les & Crawford, *Wolffia* Horkel ex Schleid. and *Wolffiella* Hegelm., with 36 different species [5]. Duckweed is considered the fastest-growing flowering plant with almost exponential growth [5]. Plant composition (e.g., crude protein or phosphorus content) differs between species and can be influenced by cultivation conditions, particularly cultivation medium [6,7]. High nutrient availability and especially ammonium and nitrate levels can increase crude protein (CP) contents by up to 44% in dry matter (DM) [8]. On the other hand, the amino acid distribution is a typical characteristic of individual species and is almost independent of environmental influences [9].

The nutritional value of duckweed has already been confirmed in several experiments with broiler chickens. However, they show some contrasting effects of using duckweed in complete diets on growth and especially feed intake [10,11]. The reasons for these effects have not yet been investigated. A possible influence could be the varying digestibility of the nutrients, in particular phosphorus (P), CP and amino acids, which has not been studied for broiler chickens yet. Tannins, which can bind proteins and cause reduced digestibility, have been detected in some studies [12,13]. However, their appearance in duckweed could not be related to any anti-nutritional effect and decreased amino acid digestibility.

The aim of this research was to investigate the effect of different duckweed batches as protein feed on zootechnical parameters, such as feed intake and body weight, as well as the ileal digestibility of amino acids and phosphorus in broiler chickens. The batches consisted of different duckweed species and are characterized by different chemical compositions. For one batch, three different ratios in the final diet were investigated.

## 2. Materials and Methods

### 2.1. Birds and Management

In total, 108 one-day-old male Ross 308 broiler chickens were obtained from Probroed & Sloot (Vreden, Germany) and raised with commercial broiler diets. On day 18, birds were transferred to individual cages (0.440 m length × 0.355 m width × 0.335 m height, floor area 0.156 m^2^) and adapted to pelleted basal feed until day 21 (Table S1). The chicks were housed in a temperature-controlled environment. An automatic system controlled temperature, humidity and ventilation, with the temperature set at 30 °C during the first week and gradually reduced to 25 °C during the second week. Artificial light was provided from 5 am until 11 pm (18L:6D). Broiler chickens and automated systems were checked twice a day. Birds had unlimited access to feed and water all the time.

On day 21, birds were weighed individually (body weight on day 21, BW21), and 18 chicks were randomly assigned to each of the 6 treatments of equal body weight for a 6-day adaption period. From day 21 to 27, all chicks were fed with the respective experimental diet as only a limited amount of duckweed biomass was available. On day 26, feces were collected from 6 birds per treatment for 24 h. On day 27, all birds were weighed again (BW27) and sacrificed by cervical dislocation. Ileal digesta was collected from the terminal half of the ileum, which was defined as part of the small intestine from Meckel’s diverticulum to approximately 4 cm anterior to the ileocecal junction. Digesta samples were subsequently stored at −20 °C in plastic containers.

### 2.2. Diets and Experimental Design

For the basal diet, a commercial broiler diet with 12.9 MJ AMEn/kg (N-corrected apparent metabolizable energy) and 18.8% CP as fed was used. The composition is given in Table S1.

The investigated duckweed batches were obtained from third parties and non-monitored production. They consisted of a mixture of *Lemna minuta* and *Lemna minor* (batch A), *Spirodela polyrhiza* (batch B) and *Lemna obscura* (batch C). They were milled with a cutting mill (3 mm matrix; Fritsch Pulverisette 25, Fritsch GmbH, Idar-Oberstein, Germany). Batches differed in their chemical composition, with CP contents of 17.5, 24.7 and 37.0% as fed for batches A, B and C, respectively. The complete nutrient composition of the individual batches is shown in Table 1 and Table S2.

Batches A and B were mixed in a share of 50% (as fed) with the basal diet (treatments A50 and B50). Batch C was mixed in proportions of 25, 50 and 75% (as fed) with the basal diet (Treatment C25, C50 and C75, respectively). Plain basal diet was fed for treatment D. For calculation of digestibility TiO_2_ was added 0.5% on top in all diets, which were pelleted with a 2.5 mm matrix size. Feed samples were taken during feed production and stored at −20 °C until analysis. The ingredients of the experimental diets and their calculated chemical composition are shown in Table 2.

### 2.3. Sample Preparation and Chemical Analyses

Species were identified by barcoding as described by Devlamynck et al. [14]. The query sequence was compared with sequences from a reference database for all duckweed species of one of the authors (M.B.).

Digesta samples were freeze-dried (P22K-E, Dieter Piatkowski—Forschungsgeräte, Petershausen, Germany) and randomly pooled to obtain 4 samples per treatment. Digesta and feed samples were milled with a centrifugal mill (UZM 200, Retsch GmbH, Haan, Germany) with a 0.2 mm sieve for Ti, P, CP and amino acid analyses. For analysis of other nutrients, feed samples were milled with a 0.5 mm sieve.

Sample preparation for P and Ti followed BVL L 00.00-19/1:2015-06 [15] and DIN EN 13805:2014-12 [16] and analysis was carried out following DIN EN ISO 17294-2:2017-01 [17].

Amino acids were analyzed after hydrolysis using chromatographic methods as described by Llames and Fontaine [18]. Nitrogen concentration was determined with a combustion method and multiplied with a factor of 6.25 for the calculation of CP contents ([19]; method 968.06).

DM, crude ash, crude fiber and crude fat were analyzed as described in annex III, letters A, M, I, H procedure B, L and J of Commission Regulation (EC) No 152/2009, respectively [20]. Some nutrients were analyzed in accordance with the methods described by VDLUFA (Verband Deutscher Landwirtschaftlicher Untersuchungs—und Forschungsanstalten e. V.) [21]: The pepsin-soluble crude protein (PSCP) was analyzed by method 4.2.1; neutral detergent fiber (NDF) by method 6.5.1; acid detergent fiber by method 6.5.2, acid detergent lignin by method 6.5.3; and calcium by method 10.8.2. Trypsin inhibitor activity (TIA) was determined in accordance to DIN EN ISO 14902:2002-02 [22], and tannin content was analyzed following the method 2.8.18(PY) of European Pharmacopoeia [23]. Inositol phosphate esters were determined according to Zeller et al. [24] using 0.5 M HCl as extractant.

Water binding capacity (WBC) was analyzed based on method 56-20 described by the American Association of Cereal Chemists [25]. Therefore, intact pellet feed samples (3.0 g ± 0.01 g) were weighed into a 50 mL centrifugal tube and mixed with 30 mL of deionized water for 20 min in a laboratory shaker (120 Hz; Laboshake, C. Gerhardt GmbH & Co. KG, Königswinter, Germany). The samples were centrifuged for 2 min at 1000× *g*. The tubes were then gently shaken to dislodge adherent particles from the lid and centrifuged again for 18 min at 1000× *g*. In the final step, the excess water was decanted and the tubes were placed at an angle of approximately 35° to drain off the remaining liquid.

### 2.4. Calculations and Statistics

In vitro digestibility (IVD) of CP was calculated as the quotient of PSCP and CP. Zootechnical parameters were trimmed to three standard deviations for all parameters (number of replicates/birds per treatment: A50, *n* = 18; B50, *n* = 17; C25, *n* = 18; C50, *n* = 17; C75, *n* = 14; C, *n* = 17). Water binding capacity was calculated as described by Serena et al. [26]. The content of AMEn was calculated as described by the WPSA [27]. The apparent ileal digestibility (AID) of individual batches was calculated by the difference method according to Nalle et al. [28]. Standardized ileal digestibility (SID) was calculated with basal endogenous losses for a nitrogen-free diet in accordance with Adeola et al. [29].

Data were statistically analyzed with SPSS (Version 26.0.0.0, IBM Corp., Armonk, NY, USA) by using the procedure UNIANOVA. The threshold of significance was set at *p* ≤ 0.05. Multiple comparison tests were performed with Sidak correction and *p* ≤ 0.05.

## 3. Results

### 3.1. Zootechnical Parameters

The highest average daily gain (ADG) and BW27 were achieved with treatment C25 (111.4 g/day), which did not significantly differ from the control group. All other feeding regimes led to a significant reduction in these parameters with the significantly lowest values for group C75 (21.6 g/day) compared to all other groups. Feed intake (FI) and average daily feed intake (ADFI) did not significantly differ between treatments A50, B50, C25 and D.

Birds of treatment C75 had the significantly lowest ADFI with 74.9 g/day, followed by group C50 with 109.3 g/day. The feed conversion ratio (FCR) was significantly reduced for treatments A50 and C75 in comparison to the control group. Group C25 had the highest FCR with 1:1.35 when compared to the other groups containing duckweed in the diet. Treatment D realized the lowest WBC (120%). With an increasing share of batch C, the dietary WBC increased to 214, 307 and 364% for treatments C25, C50 and C75. In addition, treatments A50 and B50 resulted in increased WBC (226 and 212%, respectively). Feces DM was decreased by all treatments with a share of 50% duckweed or more. Increasing shares of batch C resulted in decreasing feces DM (23.5%, 15.1% and 10.9% for treatments C25, C50 and C75, respectively). An overview of the zootechnical parameters is given in Table 3.

### 3.2. Digestibility

Apparent and standardized CP and amino acid digestibility differed significantly between batches, with batch C having the highest digestibility values compared to all other batches. The individual digestibility coefficients are given in the subsequent Table 4.

The digestibility of cysteine was the lowest within all individual batches compared to all other amino acids (see Table 4). For all amino acids and crude protein, batches consistently rank in the same order regarding the individual digestibility coefficients.

The apparent ileal digestibility of P differed significantly between batches, ranging between 50.8 and 78.9%.

## 4. Discussion

### 4.1. Nutrient Composition

The CP contents in the duckweed batches ranged from 184 to 396 g/kg DM. These values are similar to those detected by Stadtlander et al. [30] and Khanum et al. [31], who determined levels of 180 and 402 g/kg DM, respectively. The determined P contents ranged between 3.1 and 23.9 g/kg DM, which were also reported by Akter et al. [32] and Khandaker et al. [33], respectively. The varying plant composition of the investigated batches could be associated with the different species [7]. In addition, cultivation conditions, particularly the nutrient media, significantly influence the composition [6].

The amino acid contents of batches A and B are lower compared to soybean meal [34]. Batch C (*L. obscura*) has higher methionine and tryptophan contents than soybean meal with 45% CP (7.4 vs. 6.4 g methionine per kg, 7.5 vs. 5.9 g tryptophan per kg; [34]). However, the amino acid ratio is favorable for batch C because the determined contents of the limiting amino acids methionine and lysine per 100 g CP are higher than in soybean meal (2.0 vs. 1.4 g methionine/100 g CP and 6.2 vs. 6.1 g lysine/100 g CP; [34]). In the context of low-protein feeding regimens, this could reduce the requirement for synthetic amino acids.

### 4.2. Zootechnical Parameters

The poor amino acid digestibility, amino acid content, and low AMEn levels of batches A and B reduced the supply of the respective nutrients. The inclusion of these batches in the diets, therefore, caused decreased growth performance. Treatments C50 and C75 resulted in reduced feed intake causing decreased nutrient supply and growth performance. In accordance with this study, previous studies showed inconsistent influence on growth performance. Kabir et al. [10] and Islam et al. [35] tested *L. minor* with comparable crude fiber contents of 11.2 and 12.1% in DM, respectively. Increasing duckweed proportions of 4, 8 and 12% of *L. minor* led to reduced growth without any influence on feed intake [10]. However, Islam et al. [35] reported a reduced ADFI and reduced growth with proportions of 3, 6 and 9% of *L. minor*. Moreover, results of other studies show that various fiber sources and concentrations do not affect feed intake consistently [36,37]. This supports the findings in the present study. Though batches C and B had comparable NDF contents, they show different effects on feed intake, while batch A, having the highest NDF content (42% as fed) did not influence feed intake (treatment A50).

Concerning feed intake, also WBC was analyzed. Though diets A50, B50 and C25 had increased WBC (226, 212 and 214%, respectively) compared to the control (120%), ADFI did not differ significantly. WBC at those levels might not affect feed intake during short periods. Increasing shares of batch C resulted in increasing WBC and caused decreasing feed intake. The differences in WBC and ADFI between treatments A50, B50 and C50 indicate that individual batches differ regarding these parameters. It is known that WBC generally correlates with NDF fiber fraction (hemicellulose, cellulose) [38], but it does not in the present study. Consequently, the WBC of duckweed fiber could differ in the batches but also proteins and other compounds could cause the differences in WBC [39].

Duckweed biomass also influenced DM content in feces. Lower DM contents in the feces might result in higher litter moisture which can cause foot pad dermatitis, a welfare-relevant factor [40]. Fibrous compounds and higher WBC of duckweed biomass can be responsible for this increased feces moisture. Sugar beet pulp, a fiber source with a high water binding capacity, caused decreased DM content in the excretions of broiler chickens [37]. Thus, especially soluble fiber could be responsible for this effect [41]. For treatments A50 and B50 increased protein contents in feces due to lower digestibility might also increase moisture in the feces, but likewise, other substances such as K, Na or Ca can have an influence on feces DM [40]. Therefore, future studies should particularly investigate the nutritional properties of duckweed with regard to feed intake, fiber composition and water binding capacity.

### 4.3. Crude Protein and Amino Acid Digestibility

Up to now, the amino acid digestibility of duckweed has not been determined for broiler chickens. Batch C showed high amino acid digestibility values being on the same level as the microalgae *Spirulina platensis* (Lys 81.2, Met 82.4, Cys 77.8% SID; [42]) or the ensiled seaweed *Saccharina latissima* (Lys 79, Met 90, Cys 74% AID; [43]). Furthermore, the digestibility is as high as for soybean meal (Lys 90.7, Met 92.3, Cys 85,4% SID; [44]) and even higher compared to canola meal (Lys 76.9, Met 81.9, Cys 77.0% SID; [45]).

High tannin contents have been detected in the low digestible batches A and B, while low levels were measured in the highly digestible batch C. Tannins have been identified previously for duckweed in concentrations from 9 to 16 g per kg DM [12,46]. Rubanza et al. [47] determined that in vitro gas production is not only influenced by the level of tannins but also by their anti-nutritive activity. Additionally, the protein affinity for tannins varies greatly [48]. Mansoori and Acamovic [49] found a linear relationship between tannic acid dose and amino acid excretion. For example, a tannic acid dose of 6 g increased Lys excretion by approx. threefold within 48 h. Thus, variation in SID of CP and amino acid could be related to tannin contents. The negative AID of cysteine also indicates the presence of anti-nutritional factors. This is supported by the large differences between IVD and SID of CP for batches A and B, as such differences are also mainly caused by anti-nutritional factors. In vitro digestibility is not influenced by endogenous losses—especially feed-specific endogenous losses—and consequently corresponds to real digestibility [50]. However, due to the variation in IVD, it must be considered that not only tannins can affect protein digestibility. Therefore, it can be questioned whether these differences are also influenced by the different species.

Few studies have been carried out to examine the IVD of CP in duckweed, and they confirm the values found in this study. Dewanji [51] estimated 77.9% IVD for *L. minor* with 38.3% CP. Other IVD values have been found at 69% (34.4% CP in DM; [52]), 67.4% (28.5% CP in DM; [53]) and 62.2% (29.6% CP in DM; [54]) for *L. minor*. The present data also indicate that high protein contents contribute to a high IVD and high enzymatic protein accessibility.

Bond [55] identified that pepsin solubility (described as IVD in the present study) is not associated with the presence of tannins, but Kaspchak et al. [56] found a negative impact of tannic acid on IVD of bovine serum albumin at pH of 7. Thus, it remains unclear whether and to what extent the tannins contained in the respective batch influence IVD.

Individual batches rank in the same order for in vitro and standardized ileal digestibility of CP. This is consistent with Ravindran and Bryden [57] who stated that IVD of CP can provide information to rank protein sources. Therefore, this analytical parameter can be used for product quality optimization. Nevertheless, it should be considered that IVD includes solubility. Referring to Stokvis et al. [43], not all soluble nutrients are digestible. Thus, it is possible that in vitro methods overestimate real digestibility.

### 4.4. Phosphorus Digestibility

As with amino acid and CP digestibility, the P digestibility of duckweed biomass for broiler chickens has not yet been determined. Phosphorus digestibility differed significantly between the batches but was equal for different concentrations of batch C and varying Ca:P ratios. This is in line with the results of Liu et al. [58], who did not find an influence of Ca:P ratios ranging from 1.2:1 to 2:1 on P digestibility. However, there is also evidence that P digestibility decreases with wider Ca:P ratios [59]. Therefore, especially the AID of batch B with high Ca content might have been underestimated. In the investigated duckweed batches, only a small proportion of P is bound to phytate and the phytate content is low in comparison to soybean meal. Other authors, using a different analytical method, reported higher phytate contents of 12.3 and 26 g/kg DM, respectively [12,13]. At present, it is not known which factors affect the P digestibility of duckweed.

## 5. Conclusions

The tested duckweed batch of *L. obscura* can be used as an efficient protein source in proportions of up to 25% due to the high amino acid content and the high ileal digestibility of P and amino acids. Biomass had varying contents of tannins. These are known as anti-nutritive factors that can reduce the digestibility of amino acids and CP. Phosphorus digestibility was not affected by phytate due to its low concentrations. As limiting factors for adequate feed intake and growth performance, a high water binding capacity and relatively high fiber contents have been investigated. The low calculated AMEn contents of the duckweed batches and the relatively high fiber contents (NDF) should be considered in future studies concerning their impact on the nutritional value. The high variability of duckweed biomass composition and its nutritional value indicates that adequate species need to be selected and that duckweed cultivation must be optimized to provide biomass suitable for broiler chicks. A stable plant composition and a biomass production targeted to the requirements of broiler chickens are necessary to ensure adequate nutrition.

## Figures and Tables

**Table 1:** Nutrient levels of dried duckweed (g/kg as fed except where stated).

Batch	A	B	C
Species	*Lemna minuta, Lemna minor*	*Spirodela polyrhiza*	*Lemna obscura*
Dry matter	953	897	934
Crude protein	175	246	370
Ether extract	29.5	22.4	65.4
Crude fiber	124	109	123
Crude ash	142	177	56.0
AMEn	6.25	5.90	8.92
Pepsin soluble crude protein	109	168	291
In vitro digestibility (%)	61.9	68.3	78.6
Calcium	19.5	28.8	12.1
Phosphorus	9.00	5.55	5.37
Ca:P (…:1)	2.16	5.19	2.25
Phytate P	0.48	0.17	0.43
Phytate	1.72	0.59	1.52
Phytate P (% total P)	5.44	3.06	8.01
Tannin (mg/kg) ^1^	2943	2890	303
Trypsin inhibitor activity (mg/g)	*	*	*

AMEn = N-corrected apparent metabolizable energy. ^1^ As pyrogallol. * Below detection limit of 0.5 mg/kg fresh matter.

**Table 2:** Botanical and chemical composition of the experimental diets (g/kg as fed except where stated).

Treatment	A50	B50	C25	C50	C75	D
Batch A *Lemna minuta*, *Lemna minor*	500					
Batch B *Spirodela polyrhiza*		500				
Batch C *Lemna obscura*			250	500	750	
Basal diet	500	500	750	500	250	1000
TiO_2_ on top	5	5	5	5	5	5
calculated diet composition:						
Dry matter	921	893	901	911	922	890
Crude ash	99.5	117	57.2	56.7	56.2	58.2
Crude protein	181	215	232	277	322	188
Phosphorus	7.50	5.72	5.81	5.66	5.50	6.02
Calcium	14.2	18.9	9.80	10.6	11.3	9.10
Ether extract	58.5	54.9	81.9	76.3	70.7	87.7
Crude fiber	78.6	71.1	56.0	78.1	100.2	34.3
AMEn (MJ/kg as fed)	9.50	9.35	11.9	10.9	9.87	12.9
Ca:P (…:1)	1.91	3.30	1.69	1.86	2.05	1.52

AMEn = N-corrected apparent metabolizable energy.

**Table 3:** Zootechnical parameters of broiler chickens (d 21–27) and water binding capacity of experimental diets depending on the supply of duckweed.

Treatment	A50	B50	C25	C50	C75	D
Batch	A	B	C	C	C	-
WBC (% as fed) *	225.9 ± 5.0	212.4 ± 6.3	214.0 ± 3.5	307.0 ± 3.0	364.9 ± 1.9	119.6 ± 3.0
BW21 (g)	1186 ± 20	1192 ± 20	1180 ± 19	1191 ± 20	1188 ± 22	1195 ± 21
BW27 (g)	1587 ± 34 ^b^	1665 ± 32 ^b^	1848 ± 29 ^a^	1608 ±30 ^b^	1317 ± 29 ^c^	1821 ± 33 ^a^
d 21–27						
ADG (g/d)	66.9 ± 3 ^b^	78.8 ± 3.4 ^b^	111.4 ± 2.6 ^a^	69.6 ± 3.4 ^b^	21.6 ± 2.4 ^c^	104.3 ± 3.1 ^a^
FI (g)	858 ± 30 ^a^	880 ± 30 ^a^	896 ± 16 ^a^	656 ± 15 ^b^	449 ± 14 ^c^	925 ± 18 ^a^
ADFI (g/d)	143.0 ± 5.0 ^a^	146.7 ± 5.0 ^a^	149.4 ± 2.6 ^a^	109.3 ± 2.5 ^b^	74.9 ± 2.3 ^c^	154.2 ± 2.9 ^a^
FCR (1:)	2.16 ± 0.04 ^c^	1.88 ± 0.03 ^b,c^	1.35 ± 0.02 ^a^	1.61 ± 0.06 ^a,b^	3.93 ± 0.35 ^d^	1.49 ± 0.03 ^a,b^
d 26						
Feces DM (%)	15.5 ± 2.1 ^c,d^	19.4 ± 1.7 ^b,c^	23.5 ± 1.9 ^a,b^	15.1 ± 3.9 ^c,d^	10.9 ± 1.6 ^d^	26.8 ± 3.2 ^a^

WBC = water-binding capacity; BW21/BW27 = body weight on day 21/27 post hatch; ADG = average daily gain; FI = feed intake; ADFI = average daily FI; FCR = feed conversion ratio; DM = dry matter. Number of replicates/birds for zootechnical parameters and individual treatments: A50, *n* = 18; B50, *n* = 17; C25, *n* = 18; C50, *n* = 17; C75, *n* = 14; C, *n* = 17; * *n* = 2; ^abcd^ Means within the same row with different superscripts are different at *p* < 0.05.

**Table 4:** Ileal digestibility of crude protein, amino acids and P of different duckweed batches.

Batch	A	B	C	C	C
Treatment	A50	B50	C25	C50	C75
Apparent ileal digestibility (%)					
CP	34.1 ± 1.2 ^c^	55.3 ± 1.5 ^b^	79.8 ± 1.5 ^a^	80.8 ± 0.5 ^a^	78.0 ± 1.0 ^a^
Methionine	45.3 ± 2.1 ^c^	65.5 ± 2.0 ^b^	87.9 ± 1.1 ^a^	89.0 ± 0.3 ^a^	87.0 ± 0.5 ^a^
Cysteine	−7.0 ± 3.5 ^c^	27.5 ± 3.2 ^b^	73.2 ± 1.9 ^a^	76.1 ± 0.9 ^a^	75.2 ± 1.3 ^a^
Lysine	45.7 ± 1.7 ^c^	64.0 ± 2.0 ^b^	91.0 ± 1.3 ^a^	91.5 ± 0.4 ^a^	89.5 ± 0.7 ^a^
Threonine	35.2 ± 2.0 ^c^	55.2 ± 2.0 ^b^	82.1 ± 1.6 ^a^	83.8 ± 0.4 ^a^	81.7 ± 0.9 ^a^
Tryptophan	32.0 ± 2.1 ^c^	61.0 ± 1.6 ^b^	79.5 ± 1.1 ^a^	82.1 ± 0.4 ^a^	81.3 ± 0.7 ^a^
Arginine	55.2 ± 1.9 ^c^	72.9 ± 1.3 ^b^	90.6 ± 0.5 ^a^	91.5 ± 0.5 ^a^	91.0 ± 1.0 ^a^
Isoleucine	45.0 ± 2.4 ^c^	63.4 ± 1.9 ^b^	85.8 ± 1.3 ^a^	87.4 ± 0.4 ^a^	85.3 ± 0.7 ^a^
Leucine	48.0 ± 1.9 ^c^	65.8 ± 1.7 ^b^	88.0 ± 1.1 ^a^	88.8 ± 0.3 ^a^	86.7 ± 0.6 ^a^
Valine	44.1 ± 2.4 ^c^	62.0 ± 1.6 ^b^	86.1 ± 1.3 ^a^	87.7 ± 0.4 ^a^	85.7 ± 0.7 ^a^
Histidine	28.1 ± 3.0 ^c^	50.1 ± 2.4 ^b^	84.9 ± 1.6 ^a^	86.5 ± 0.6 ^a^	84.3 ± 0.8 ^a^
Phenylalanine	47.4 ± 1.9 ^c^	66.5 ± 1.5 ^b^	87.4 ± 1.1 ^a^	88.7 ± 0.3 ^a^	86.8 ± 0.5 ^a^
Glycine	31.7 ± 1.8 ^c^	51.6 ± 1.4 ^b^	79.0 ± 1.5 ^a^	80.6 ± 0.5 ^a^	78.9 ± 0.7 ^a^
Serine	29.3 ± 2.2 ^c^	54.6 ± 2.0 ^b^	79.2 ± 2.0 ^a^	80.9 ± 0.3 ^a^	79.7 ± 0.9 ^a^
Proline	24.2 ± 1.9 ^c^	45.1 ± 2.1 ^b^	82.1 ± 1.0 ^a^	84.0 ± 0.4 ^a^	83.1 ± 0.9 ^a^
Alanine	48.0 ± 1.8 ^c^	64.8 ± 1.5 ^b^	86.6 ± 1.4 ^a^	87.6 ± 0.2 ^a^	85.0 ± 0.6 ^a^
Asparagine	43.4 ± 1.7 ^c^	67.3 ± 1.6 ^b^	85.6 ± 1.4 ^a^	87.8 ± 0.4 ^a^	86.0 ± 0.8 ^a^
Glutamine	31.5 ± 2.5 ^c^	54.6 ± 2.0 ^b^	86.6 ± 1.4 ^a^	88.6 ± 0.5 ^a^	86.1 ± 0.8 ^a^
Sum AA	39.2 ± 1.9 ^c^	60.3 ± 3.8 ^b^	85.2 ± 1.3 ^a^	86.7 ± 0.4 ^a^	84.8 ± 0.7 ^a^
P	78.9 ± 1.2 ^a^	50.8 ± 1.8 ^c^	67.8 ± 2.0 ^b^	68.1 ± 2.0 ^b^	63.7 ± 1.0 ^b^
Standardized ileal digestibility (%)					
CP	40.2 ± 1.2 ^c^	59.6 ± 1.5 ^b^	82.7 ± 1.5 ^a^	83.7 ± 0.5 ^a^	80.9 ± 1 ^a^
Methionine	49.9 ± 2.1 ^c^	68.5 ± 2.0 ^b^	89.3 ± 1.1 ^a^	90.4 ± 0.3 ^a^	88.4 ± 0.5 ^a^
Cysteine	16.9 ± 3.5 ^c^	42.5 ± 3.2 ^b^	82.6 ± 1.9 ^a^	85.6 ± 0.9 ^a^	84.6 ± 1.3 ^a^
Lysine	51.1 ± 1.7 ^c^	67.4 ± 2.0 ^b^	92.5 ± 1.3 ^a^	93.1 ± 0.4 ^a^	91.1 ± 0.7 ^a^
Threonine	44.6 ± 2.0 ^c^	61.4 ± 2.0 ^b^	85.7 ± 1.6 ^a^	87.3 ± 0.4 ^a^	85.3 ± 0.9 ^a^
Tryptophan	35.5 ± 2.1 ^c^	63.3 ± 1.6 ^b^	80.6 ± 1.1 ^a^	83.2 ± 0.4 ^a^	82.4 ± 0.7 ^a^
Arginine	60.1 ± 1.9 ^c^	75.6 ± 1.3 ^b^	92.2 ± 0.5 ^a^	93.1 ± 0.5 ^a^	92.6 ± 1.0 ^a^
Isoleucine	50.7 ± 2.4 ^c^	67.3 ± 1.9 ^b^	87.8 ± 1.3 ^a^	89.4 ± 0.4 ^a^	87.3 ± 0.7 ^a^
Leucine	52.9 ± 1.9 ^c^	69.1 ± 1.7 ^b^	89.7 ± 1.1 ^a^	90.4 ± 0.3 ^a^	88.4 ± 0.6 ^a^
Valine	50.5 ± 2.4 ^c^	66.0 ± 1.6 ^b^	88.4 ± 1.3 ^a^	89.9 ± 0.4 ^a^	87.9 ± 0.7 ^a^
Histidine	35.5 ± 3.0 ^c^	54.7 ± 2.4 ^b^	87.1 ± 1.6 ^a^	88.7 ± 0.6 ^a^	86.4 ± 0.8 ^a^
Phenylalanine	52.4 ± 1.9 ^c^	69.9 ± 1.5 ^b^	89.1 ± 1.1 ^a^	90.4 ± 0.3 ^a^	88.5 ± 0.5 ^a^
Glycine	37.4 ± 1.8 ^c^	55.6 ± 1.4 ^b^	81.3 ± 1.5 ^a^	82.9 ± 0.5 ^a^	81.1 ± 0.7 ^a^
Serine	37.8 ± 2.2 ^c^	60.3 ± 2.0 ^b^	82.5 ± 2.0 ^a^	84.2 ± 0.3 ^a^	82.9 ± 0.9 ^a^
Proline	31.5 ± 1.9 ^c^	50.2 ± 2.1 ^b^	84.8 ± 1.0 ^a^	86.8 ± 0.4 ^a^	85.9 ± 0.9 ^a^
Alanine	52.3 ± 1.8 ^c^	67.6 ± 1.5 ^b^	88.3 ± 1.4 ^a^	89.2 ± 0.2 ^a^	86.6 ± 0.6 ^a^
Asparagine	48.2 ± 1.7 ^c^	69.8 ± 1.6 ^b^	87.7 ± 1.4 ^a^	89.9 ± 0.4 ^a^	88.1 ± 0.8 ^a^
Glutamine	37.8 ± 2.5 ^c^	58.5 ± 2.0 ^b^	89.0 ± 1.4 ^a^	91.0 ± 0.5 ^a^	88.6 ± 0.8 ^a^
Sum AA	45.2 ± 1.9 ^c^	64.1 ± 1.7 ^b^	87.4 ± 1.3 ^a^	88.9 ± 0.4 ^a^	87.0 ± 0.7 ^a^

CP = crude protein; AA = amino acid. ^abc^ Means within the same row with different superscripts are different at *p* < 0.05. *n* = 4 (pooled samples of 4 to 5 birds in each).

## Data Availability

The data presented in this study can be provided on request.

## Supplementary Materials

The following supporting information can be downloaded at: https://www.mdpi.com/article/10.3390/ani13010130/s1, Table S1: Botanical composition of the basal diet; Table S2: Fiber composition and amino acid profile of dried duckweed (g/kg as fed except where stated).
