# Supplementary material for: Nutritional Value of Duckweed as Protein Feed for Broiler Chickens—Digestibility of Crude Protein, Amino Acids and Phosphorus
Source: Animals (Basel). 2022 Dec 29;13(1):130. doi: 10.3390/ani13010130 (PMC9817926; doi:10.3390/ani13010130)
Supplement: Supplementary file 1 [file animals-13-00130-s001.zip › Table S1.pdf]

Table S1. Botanical composition of the basal diet

Ingredients (g/kg)	Basal diet
Maize	433.5
Wheat	150.0
Soybean meal (46 % CP)	261.0
Rapeseed meal	60.0
Vegetable oil (non GMO)	59.5
Limestone ($CaCO_3$)	12.3
L-Lysine (78%)	3.0
L-Threonine	0.9
L-Methionine	2.6
Mono-calcium-phosphate (MCP)	9.0
Salt ($NaHCO_3$+NaCl)	3.0
Premix (Vit./Min.)[1]	5.2

[1] feed additives per kg as fed: vitamin A (3a672a) 10000 IU, vitamin D3 (3a671) 4000 IU, vitamin E (all-rac-alpha-tocopherylacetate) (3a700) 38 IU, selenium (3b801, Na-selenite) 0.34 mg, copper (3b405, Cu(II)-sulfate, pentahydrate) 6.5 mg, copper (3b4.10, Cu-chelate of methionine hydroxy-analogue) 8.3 mg, zinc (3b603, Zn-oxide) 31.2 mg, zinc (3b6.10, Zn-chelate of methionine hydroxy-analogue) 34.6 mg, manganese (3b502, Mn-(II)-oxide) 20.8 mg, manganese (3b5.10, Mn-chelate of methionine hydroxy-analogue) 34.6 mg, iron (3b103, Fe-(II)-sulfate, monohydrate) 76.6 mg, iodine (3b202, Ca-iodate) 1.1 mg, calcium salt of methionine hydroxy-analogue 2.23 g, 6-Phytase EC 3.1.3.26 (Quantum Blue) (4a19) 260 FTU, Endo-1,4-Beta-Xylanase EC 3.2.1.8 (4a15) 610 U, Endo 1,3 (4)-ß-Glucanase EC 3.2.1.6 (4al5) 76 U, Butylated hydroxytoluene (E321) 60.8 mg, sepiolite (E 562) 0.3 mg
