# Supplementary material for: Nutritional Value of Duckweed as Protein Feed for Broiler Chickens—Digestibility of Crude Protein, Amino Acids and Phosphorus
Source: Animals (Basel). 2022 Dec 29;13(1):130. doi: 10.3390/ani13010130 (PMC9817926; doi:10.3390/ani13010130)
Supplement: Supplementary file 1 [file animals-13-00130-s001.zip › Table S2.pdf]

Table S2. Fiber composition and amino acid profile of dried duckweed (g/kg as fed except where stated)

**Batch**	**A**	**B**	**C**
Species	*Lemna minuta, Lemna minor*	*Spirodela polyrhiza*	*Lemna obscura*
Dry matter	953	897	934
Crude fiber	124	109	123
Neutral detergent fiber	421	275	294
Acid detergent fiber	200	234	186
Acid detergent lignin	30.5	56.5	29.0
Crude protein	175	246	370
Methionine	2.26	3.26	7.42
Cysteine	1.63	2.45	4.06
Lysine	6.88	10.20	22.87
Threonine	6.14	8.76	15.88
Tryptophan	2.45	3.47	7.54
Arginine	7.58	12.97	22.98
Isoleucine	6.23	8.46	17.05
Leucine	10.94	15.31	31.67
Valine	7.50	11.33	21.24
Histidine	2.33	3.49	7.78
Phenylalanine	7.08	9.76	19.85
Glycine	7.90	10.55	19.51
Serine	6.29	8.78	16.07
Proline	6.48	8.87	16.90
Alanine	8.57	12.40	21.70
Asparagine	14.51	26.44	32.41
Glutamine	14.90	22.73	37.74
